# ﻿Contributions to the knowledge of pitvipers (Viperidae, *Gloydius*) in the Democratic People’s Republic of Korea: identification, description of specimens, and geographical distribution

**DOI:** 10.3897/zookeys.1249.142916

**Published:** 2025-08-19

**Authors:** Yucheol Shin, Siti N. Othman, Dallin B. Kohler, Irina Maslova, Kevin R. Messenger, Amaël Borzée

**Affiliations:** 1 Laboratory of Animal Behaviour and Conservation, College of Life Sciences, Nanjing Forestry University, Nanjing 210037, China; 2 Richard Gilder Graduate School, American Museum of Natural History, Central Park West at 79th Street, New York, NY 10024, USA; 3 Department of Herpetology, American Museum of Natural History, Central Park West at 79th Street, New York, NY 10024, USA; 4 Laboratory of Animal Behaviour and Conservation, College of Ecology and the Environment, Nanjing Forestry University, Nanjing 210037, China; 5 Federal Scientific Centre of the East Asia Terrestrial Biodiversity, Far Eastern Branch of Russian Academy of Sciences, Russia; 6 Herpetology and Applied Conservation Lab, College of Life Sciences, Nanjing Forestry University, Nanjing 210037, China; 7 Department Zoology and General Biology, Faculty of Life Sciences, Fergana State University, Fergana 150100, Uzbekistan

**Keywords:** Ecological niche modeling, *Gloydius*, Korea, morphological analyses, phylogenetic analyses, venomous snakes

## Abstract

Basic knowledge of species distribution and diversity is crucial for broader studies of ecology, evolutionary biology, and conservation. However, a basic inventory of species diversity is difficult to obtain in nations that are inaccessible for fieldwork, such as the Democratic People’s Republic of Korea (DPR Korea). The diversity and distribution of snake fauna of DPR Korea are described in only a few publications, and no physical specimens have been examined recently. Here, the first detailed descriptions of pitvipers of the genus *Gloydius* from DPR Korea are provided, based on four specimens purchased from a market as “snake liquor.” Morphological and genetic data were analyzed to identify these specimens at the species level. Ecological niche modeling was also implemented to estimate the geographical distributions of *Gloydius* within DPR Korea. Phylogenetic analyses of the mitochondrial 12S rRNA gene fragments identified the samples to three species: *G.ussuriensis*, *G.brevicauda*, and *G.intermedius*. The results of multivariate morphological analyses and diagnostic scalation and body patterns were also congruent with molecular identifications. The niche models had high predictive performance and predicted suitable habitats that were consistent with the ecology of the three species. With this study, the first morphological and genetic data for *Gloydius* from DPR Korea is provided that can be incorporated into future studies. In addition, the predicted distribution of *Gloydius* within DPR Korea can be used to map the snakebite risk in the nation. Our study adds to the efforts towards a better understanding of the herpetofauna of DPR Korea.

## ﻿Introduction

Knowledge of the species diversity and distribution provides necessary data for broader studies in ecology, evolutionary biology, and conservation. The availability of such data has increased dramatically in recent years thanks to publicly available biodiversity databases, increased survey efforts, rapid developments in analytical methods, and citizen science (Heberling et al. 2021; Sahu et al. 2023). While such developments have led to an increased understanding of biodiversity in poorly studied regions, several nations still remain challenging or largely inaccessible to field surveys and biodiversity research (Borzée et al. 2021; Jablonski et al. 2023).

In this regard, the Democratic People’s Republic of Korea (DPR Korea hereafter) is among the least explored nations for biodiversity. The terrestrial fauna of DPR Korea is poorly documented and the reptile fauna of DPR Korea in particular is reported in only a small number of publications (Stejneger 1907; Shannon 1956; Won 1971; Lee and Gil 2007; Kim and Han 2009). Most of these publications include this information as part of broader faunal studies or provide only general accounts for each reported species. Nevertheless, recent studies have contributed significantly to the knowledge of the herpetofauna in DPR Korea. For example, Borzée et al. (2021) provided a thorough and comprehensive review of amphibians in the nation, combining field surveys, molecular analyses, call recording, niche modeling, photographic identification, and a literature review. However, such a review is not available for reptile species. The reptile fauna of DPR Korea is likely to be similar to that of surrounding regions, such as the Far East of the Russian Federation (Russia), the People’s Republic of China (PR China), and the Republic of Korea (R Korea) due to the geographic proximity. However, several species with unclear taxonomic identity and distribution are known from DPR Korea, including “*Eumecescoreeensis*,” *Scincellahuanrenensis*, “*Elaphecoreana*,” and *E.taeniura* (Stejneger 1907; Won 1971; Kim and Han 2009; Shin et al. 2021a). Detailed data for the herpetofauna of DPR Korea overall help to fill the gaps in our knowledge of their distribution and ecology, and aid downstream research such as transboundary range predictions and conservation assessments (Shin et al. 2021a; Borzée et al. 2021; Li et al. 2022; Peng et al. 2023).

**Figure 1.**
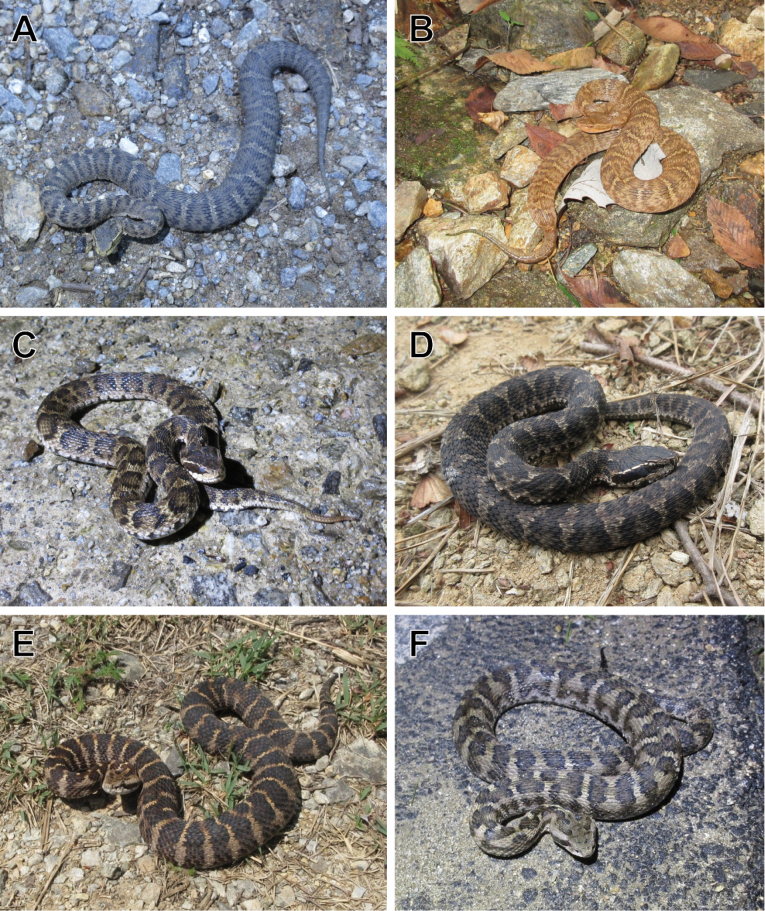
Representative photographs of the three *Gloydius* species from the Korean Peninsula in life. A. *G.ussuriensis* from Hwacheon, Gangwon Province, R Korea; B. *G.ussuriensis* from Hongcheon, Gangwon Province, R Korea; C. *G.brevicauda* from Yanggu, Gangwon Province, R Korea; D. *G.brevicauda* from Taean, South Chungcheong Province, R Korea; E. *G.intermedius* from Yeongwol, Gangwon Province, R Korea; F. *G.intermedius* from Chuncheon, Gangwon Province, R Korea. Photographs by YS.

**Figure 2.**
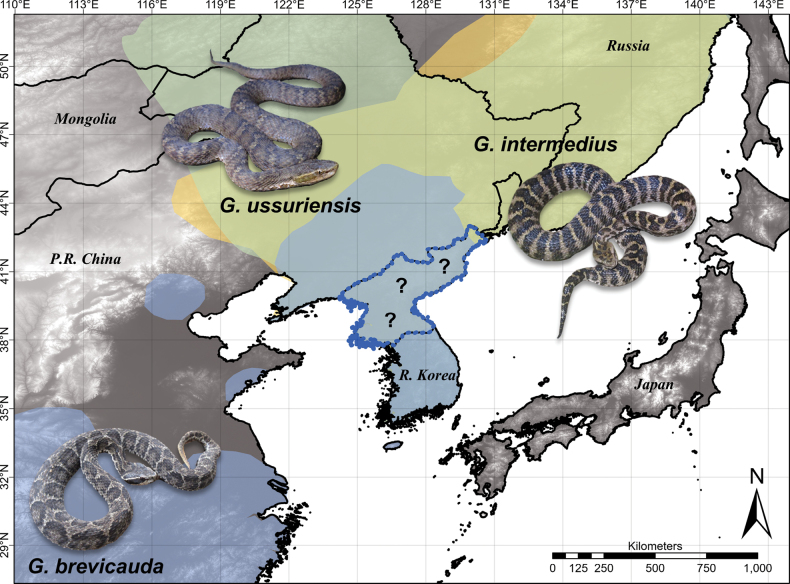
The overall geographical distributions of *Gloydiusussuriensis* (green), *G.brevicauda* (blue), and *G.intermedius* (orange) in continental northeast Asia. The defined ranges for *G.ussuriensis* and *G.intermedius* are based on the range polygons obtained from the IUCN Red List of Threatened Species (https://www.iucnredlist.org/). The range map for *G.brevicauda* is based on the combination of iNaturalist and GBIF data, IUCN range map, and personal data of KRM. The location of DPR Korea is outlined in blue. The question marks across DPR Korea denote the uncertain distributions of *Gloydius* species within the nation. The map was made in ArcGIS Pro v. 2.6.0 with the WorldClim 1 km elevation raster as a base map. This map is not meant to be a precise range map for these species but rather a general representation of their geographic distributions around DPR Korea. The inset photographs of three *Gloydius* species were taken by YS.

In this study, we focus on the snake genus *Gloydius*, which is a genus of medically significant pitvipers with 25 currently described species (Uetz et al. 2024). Members of the genus are widespread across eastern and central Asia. Three species (*G.brevicauda* (Stejneger, 1907), *G.intermedius* (Strauch, 1868), and *G.ussuriensis* (Emelianov, 1929); Fig. 1) are known from regions adjacent to DPR Korea (R Korea, northeastern China, and the Russian Far East; Orlov and Barabanov 1999; Lee et al. 2023; Uetz et al. 2024; Fig. 2). Therefore, the presence of at least these three species within DPR Korea is suspected due to geographic proximity. The descriptions of *Gloydius* from DPR Korea primarily come from Won (1971) and Kim and Han (2009). However, the taxonomy and descriptions provided therein are insufficient to make species-level identifications under the current taxonomy of the genus. In addition, the geographic distributions provided in these publications are mostly given at the province level, making it difficult to map the detailed distributions of these species in DPR Korea. Furthermore, to our knowledge, there are no recently collected vouchers of *Gloydius* from DPR Korea.

Here, we examine four preserved specimens of *Gloydius* from DPR Korea. Using morphological and molecular data, we identify these specimens to the species level and map their geographic distributions within DPR Korea using ecological niche modeling. In doing so, we provide the most comprehensive overview of this genus for this nation to date.

## ﻿Materials and methods

### ﻿Specimen collection from DPR Korea

The specimens we examined in this study are four *Gloydius* specimens collected from DPR Korea during fieldwork conducted in 2019 by AB. These specimens were legally purchased as “snake liquor” at a local food market in Pyeongyang, DPR Korea. Commercial importation of snakes into DPR Korea from other countries is not known to take place and highly unlikely considering the status of this nation, and snakes are frequently collected locally for human consumption in DPR Korea (Kim and Han 2009). In addition, AB directly inquired the sellers regarding the geographical provenance of the snakes (see below). Thus, we are confident that the snakes examined in this study were collected within DPR Korea. We subsequently transferred the specimens from the original containers to glass bottles filled with 95% ethanol, assigned the voucher numbers 19GNK001, 19GNK002, 19GNK003, and 19GNK004 to the specimens, and deposited them in the Laboratory of Animal Behaviour and Conservation, Nanjing Forestry University, PR China. According to the sellers, voucher 19GNK001 was collected from Rajin, DPR Korea, voucher 19GNK002 was collected from an unspecified location in DPR Korea, and vouchers 19GNK003 and 19GNK004 were collected from the vicinity of Gaesong, DPR Korea. While we are confident that the specimens are from DPR Korea, we did not include them in spatial analyses due to the imprecise locality information (see below).

### ﻿Morphological identification

We recorded the following diagnostic scalation characteristics based on the definitions in Zhao (2006) to identify the four *Gloydius* specimens collected from DPR Korea. 1) The number of supralabials (supralabials): count of scales along the upper lip, not including the rostral (counted for both sides of the head). 2) The number of infralabials (infralabials): count of scales along the lower lip, not including the mental (counted for both sides of the head). 3) The number of dorsal scale rows counted at mid-body. 4) The number of ventral scales: count of large scales on the ventral surface anterior to the anal plate. 5) The number of subcaudal scales: count of scales posterior to the anal plate and anterior to the keratinous tail tip. As *Gloydius* has divided subcaudal scales, the subcaudal counts represent the number of paired scales. For species diagnosis based on scalation, we also consulted Paik and Yang (1989), Orlov and Barabanov (2000), Do et al. (2019), and Lee et al. (2023).

In addition, we measured nine morphometric characters using the following definitions. 1) Snout-vent length (**SVL**): measured from the tip of the snout to the anterior edge of the vent. 2) Tail length (**TAL**): from the anterior edge of the vent to the tip of the tail. 3) Total length (**TOL**): sum of SVL and TAL. 4) Head length (**HL**): measured from the tip of the snout to the jaw joint. 5) Head width (**HW**): measured at the widest point of the head close to the jaw joint. 6) Head height (**HH**): measured as perpendicular length at the tallest point. 7) Eye diameter (**ED**): measured from the most anterior corner of the eye to the most posterior corner. 8) Interorbital distance (**IOD**): measured at the widest point between eyes. 9) Internarial distance (**IN**): distance between the two nares. In addition to the raw measurements, we scaled the measured values to total body length to limit allometric effects (Wang et al. 2019). We used total body length instead of head width or head length to scale these variables because evident physical distortions of some specimens (e.g., voucher 19GNK002) related to preservation may introduce biases when scaled to head measurements. Using total length instead of SVL was appropriate, as all the examined specimens had intact tails. The definitions of morphometric characters are based on Shi et al. (2017) and Wang et al. (2019). The SVL and TAL were measured to the nearest mm using a measuring tape, and all the head measurements were taken with digital calipers (Insize, Suzhou, China) to the nearest 0.1 mm. Prior to the analyses, we tentatively identified the vouchers 19GNK001 and 19GNK004 as *G.ussuriensis*, 19GNK003 as *G.brevicauda*, and 19GNK002 as *G.intermedius* based on visual examinations of head and body patterns.

For comparative materials, we measured the same morphometric characteristics from two preserved specimens of *G.intermedius* collected from the Primorsky Territory, Russia. These samples are stored in the Bioresource Collection of the Federal Scientific Centre of East Asia Terrestrial Biodiversity of the Far East Branch of the Russian Academy of Sciences (reg. number 2797657). In addition, we accessed historical vouchers of *G.ussuriensis*, *G.brevicauda*, and *G.intermedius* collected from R Korea and deposited in the Ewha Womans University Natural History Museum, R Korea (EWNHM; Shin et al. 2020). For these specimens, we measured the above morphometric characteristics from photographs of specimens using ImageJ (Schneider et al. 2012). The protocol for photography is outlined in Shin et al. (2020). We also incorporated the morphometric measurements provided by Qiu et al. (2023) for *G.intermedius* collected from PR China to maximize the number of available samples for the analyses. Thus, we included a total of 13 *G.ussuriensis*, six *G.brevicauda*, and 12 *G.intermedius* for the multivariate morphological analyses (Table 1). We did not separate the males and females for the analyses as the goals were interspecific comparisons and species-level identifications. Furthermore, we did not include scale counts in the multivariate analyses outlined below.

**Table 1.** A list of *Gloydius* specimens used for morphological comparisons.

Species	Voucher	Locality	Source
*Gloydiusussuriensis*	19GNK001	Rajin, DPR Korea	This study
*Gloydiusintermedius*	19GNK002	DPR Korea	This study
*Gloydiusbrevicauda*	19GNK003	Gaesong, DPR Korea	This study
*Gloydiusussuriensis*	19GNK004	Gaesong, DPR Korea	This study
*Gloydiusussuriensis*	EWNHM-ANIMAL-6518	Mujugucheondong, R Korea	Shin et al. 2020
*Gloydiusussuriensis*	EWNHM-ANIMAL-6521	Palbongsan, Hongcheon, R Korea	Shin et al. 2020
*Gloydiusussuriensis*	EWNHM-ANIMAL-6522	Balang-ri, Paju, R Korea	Shin et al. 2020
*Gloydiusussuriensis*	EWNHM-ANIMAL-6547	Jinburyeong, R Korea	Shin et al. 2020
*Gloydiusussuriensis*	EWNHM-ANIMAL-6548	Hwajeon, R Korea	Shin et al. 2020
*Gloydiusussuriensis*	EWNHM-ANIMAL-6550	Gwangneung, R Korea	Shin et al. 2020
*Gloydiusussuriensis*	EWNHM-ANIMAL-6554	Jeju, R Korea	Shin et al. 2020
*Gloydiusussuriensis*	EWNHM-ANIMAL-6555	Mujugucheondong, R Korea	Shin et al. 2020
*Gloydiusussuriensis*	EWNHM-ANIMAL-6556	Mujugucheondong, R Korea	Shin et al. 2020
*Gloydiusussuriensis*	EWNHM-ANIMAL-6560	Gwangneung, R Korea	Shin et al. 2020
*Gloydiusussuriensis*	EWNHM-ANIMAL-6561	Gwangneung, R Korea	Shin et al. 2020
*Gloydiusbrevicauda*	EWNHM-ANIMAL-6501	Yeongju, R Korea	Shin et al. 2020
*Gloydiusbrevicauda*	EWNHM-ANIMAL-6505	Gwangpan-ri, Chuncheon, R Korea	Shin et al. 2020
*Gloydiusbrevicauda*	EWNHM-ANIMAL-6520	Yangju, R Korea	Shin et al. 2020
*Gloydiusbrevicauda*	EWNHM-ANIMAL-6551	Godaedo, R Korea	Shin et al. 2020
*Gloydiusbrevicauda*	EWNHM-ANIMAL-6552	Yukgokcheon, Euiseong, R Korea	Shin et al. 2020
*Gloydiusintermedius*	N1	Primorsky Territory, Russia	This study
*Gloydiusintermedius*	N2	Primorsky Territory, Russia	This study
*Gloydiusintermedius*	EWNHM-ANIMAL-6506	Daegwanryeong, R Korea	Shin et al. 2020
*Gloydiusintermedius*	SYNU040271	Kuandian, Liaoning, PR China	Qiu et al. 2023
*Gloydiusintermedius*	SYNU040272	Kuandian, Liaoning, PR China	Qiu et al. 2023
*Gloydiusintermedius*	SYNU040273	Kuandian, Liaoning, PR China	Qiu et al. 2023
*Gloydiusintermedius*	SYNU040274	Kuandian, Liaoning, PR China	Qiu et al. 2023
*Gloydiusintermedius*	SYNU040275	Kuandian, Liaoning, PR China	Qiu et al. 2023
*Gloydiusintermedius*	SYNU040276	Kuandian, Liaoning, PR China	Qiu et al. 2023
*Gloydiusintermedius*	SYNU040277	Kuandian, Liaoning, PR China	Qiu et al. 2023
*Gloydiusintermedius*	SYNU040278	Kuandian, Liaoning, PR China	Qiu et al. 2023

We first conducted a principal components analysis (PCA) with a varimax rotation to visualize the morphological clustering of the three different *Gloydius* species and to locate the positions of the three samples originating from DPR Korea within the defined morphospace. As SVL and TAL values were missing for some specimens, we excluded these variables from the PCA. We also removed highly correlated variables with Pearson’s | *r* | > 0.8 prior to the analysis. We then natural log-transformed the data and scaled the values of variables to have unit variance. We retained the principal components (PCs) with eigenvalues greater than 1, following the Kaiser criterion. We further investigated the morphological clustering of our samples by conducting a discriminant analysis of principal components (DAPC; Jombart et al. 2010) on the same dataset used for the PCA. For the DAPC, we assigned *a priori* group membership to each sample based on the known species-level identification (for museum vouchers) and tentative identification (for specimens from DPR Korea). The DAPC was conducted using the *adegenet* package (Jombart 2008). The analyses and data visualization were conducted in R v. 4.2.2 (R Core Team 2022).

### ﻿Laboratory protocols

Considering that the specimens were discolored, distorted, and degraded from preservation, we leveraged genetic data to confirm the initial morphological species identification. We sampled muscle tissues from each specimen and extracted genomic DNA using the Qiagen Blood & Tissue kit (Qiagen, Germany) following the manufacturer’s protocols. We initially selected the following mitochondrial loci for analyses: cytochrome *b* (cyt*b*), cytochrome *c* oxidase subunit 1 (COI), NADH dehydrogenase subunit 2 (ND2), NADH dehydrogenase subunit 4 (ND4), 12S ribosomal RNA (12S rRNA), and 16S ribosomal RNA (16S rRNA). The primer pairs used for these loci were: L14919 and H16064 for cyt*b* (Burbrink et al. 2000), COI(+)deg1 and COI(-)bdeg for COI (Utiger et al. 2002), L4437b and tRNA-trpR for ND2 (Ashton and de Queiroz 2001), ND4 and Leu for ND4 (Arévalo et al. 1994), L1091mod and H1557mod for 12S rRNA (Zaher et al. 2009), 16Sar-L and 16Sbr-H for 16S rRNA (Palumbi et al. 1991).

We then amplified these loci using the polymerase chain reaction (PCR). The PCR reactions were prepared in a 40 μL total volume containing 20 μL of 2x T8 High-Fidelity Master Mix (Tsingke Biotech, China), 2 μM of each forward and reverse primer, with final concentration of 0.2 μM for both, 2 μM of template DNA, and 14 μL of nuclease-free water. We followed the thermal cycling protocol for each gene as described in the references for the respective primer pairs (see above). The purified PCR products were sequenced on an ABI 3730 DNA Analyzer (Applied Biosystems, USA) at Tsingke Biotech (Beijing, China) using the same primer pairs. All loci other than 12S rRNA either failed to amplify at the PCR stage, or resulted in highly degraded, poor-quality sequences that were unsuitable for phylogenetic analyses. We successfully sequenced fragments of 12S rRNA gene with sufficient quality from the samples collected from DPR Korea, except for the voucher 19GNK004 that failed to amplify at the PCR step. Therefore, we conducted downstream phylogenetic analyses with the 12S rRNA gene only. All newly generated 12S rRNA sequences were deposited in GenBank under the accession numbers PQ638929–PQ638931 (Table 2).

**Table 2.** Sampling of the mitochondrial 12S ribosomal RNA (12S rRNA) gene for phylogenetic analyses. The GenBank accession numbers of newly generated sequences are in bold.

Species	Voucher	Locality	Accession No.	Source
*Gloydiusussuriensis*	19GNK001	Rajin, DPR Korea	**PQ638929**	This study
*Gloydiusintermedius*	19GNK002	DPR Korea	**PQ638930**	This study
*Gloydiusbrevicauda*	19GNK003	Gaesong, DPR Korea	**PQ638931**	This study
*Gloydiusussuriensis*	–	Heilongjang, PR China	NC026553	–
*Gloydiusussuriensis*	CHS318	Jilin, PR China	MK065475	Li et al. 2020
*Gloydiusussuriensis*	–	Heilongjang, PRChina	KP262412	–
*Gloydiusussuriensis*	CHS092	Liaoning, PRChina	MK065346	Li et al. 2020
*Gloydiusussuriensis*	CHS316	Jilin, PRChina	MK065474	Li et al. 2020
*Gloydiusussuriensis*	NIBRRP0000100322	Jeju, R Korea	JQ815357	Jeong et al. 2013
*Gloydiusussuriensis*	NIBRRP0000100352	Sacheon, R Korea	JQ815355	Jeong et al. 2013
*Gloydiusussuriensis*	NIBRRP0000100367	Incheon, R Korea	JQ815356	Jeong et al. 2013
*Gloydiusbrevicauda*	CHS309	Liaoning, PR China	MK065473	Li et al. 2020
*Gloydiusbrevicauda*	CHS086	Jiangsu, PR China	MK065341	Li et al. 2020
*Gloydiusbrevicauda*	CHS308	Anhui, PR China	MK065472	Li et al. 2020
*Gloydiusbrevicauda*	CHS744	Hunan, PR China	MK065614	Li et al. 2020
*Gloydiusbrevicauda*	CHS804	Hubei, PR China	MK065665	Li et al. 2020
*Gloydiusbrevicauda*	NIBRRP0000100264	Sinan, R Korea	JQ815352	Jeong et al. 2013
*Gloydiusbrevicauda*	NIBRRP0000100276	–	JQ815353	Jeong et al. 2013
*Gloydiusbrevicauda*	NIBRRP0000100265	Sinan, R Korea	JQ815354	Jeong et al. 2013
*Gloydiusintermedius*	NNU95050	Hohhot, PR China	EF012806	–
*Gloydiusintermedius*	NNU95067	Liaoning, PR China	EF012808	–
*Gloydiusintermedius*	NNU95068	Liaoning, PR China	EF012809	–
*Gloydiusintermedius*	CHS324	Liaoning, PR China	MK065478	Li et al. 2020
*Gloydiusintermedius*	CHS327	Jilin, PR China	MK065480	Li et al. 2020
*Gloydiusintermedius*	CHS322	Shaanxi, PR China	MK065476	Li et al. 2020
*Gloydiusintermedius*	CHS323	Shaanxi, PR China	MK065477	Li et al. 2020
*Gloydiusintermedius*	NIBRRP0000100251	Hwaseong, R Korea	JQ815350	Jeong et al. 2013
*Gloydiusintermedius*	NIBRRP0000100199	Samcheok, R Korea	JQ815351	Jeong et al. 2013
*Gloydiushimalayanus*	–	India	NC068353	–
*Gloydiushimalayanus*	–	India	MK559438	–
*Protobothropsmangshanensis*	KIZ014468	PR China	MW133315	–

### ﻿Phylogenetic analyses

We collected additional 12S rRNA gene sequence data of *G.ussuriensis*, *G.brevicauda*, and *G.intermedius* from GenBank (https://www.ncbi.nlm.nih.gov/genbank/; accessed on 27 December 2023) for phylogenetic analyses. These sequences mostly originated from individuals collected in R Korea and PR China (Jeong et al. 2013; Li et al. 2020). For outgroups, we used *G.himalayanus* and *Protobothropsmangshanensis* based on previous studies on the phylogeny of *Gloydius* (Xu et al. 2012; Shi et al. 2021). The GenBank accession numbers for both ingroup and outgroup taxa are listed in Table 2. Next, we aligned the sequences using MUSCLE v. 5.1 (Edgar 2004) with default settings, implemented in Geneious Prime (v. 2023.2.1; Biomatters Ltd., Auckland, New Zealand). We trimmed both ends of the aligned sequences to minimize missing data. The final alignment was composed of 31 sequences of the 12S rRNA gene fragments, each 351 bp long.

We used PartitionFinder v. 2.1.1 (Lanfear et al. 2017) to determine the best partitioning scheme and best-fit nucleotide substitution model for the sequence alignment of the 12S rRNA gene fragment. We ran the analysis with the greedy search algorithm (Lanfear et al. 2012), unlinked branch lengths, and selected the best-fit nucleotide substitution model based on the Bayesian Information Criterion (BIC). The best partitioning scheme and model were a single partition for the 12S rRNA alignment with a HKY model with a gamma-distributed rate (HKY + G). We then conducted phylogenetic analyses using both Bayesian Inference (BI) and Maximum Likelihood (ML) methods. To construct the BI tree, we used MrBayes v. 3.2.7 (Ronquist et al. 2012). We conducted two independent runs of Markov Chain Monte Carlo (MCMC) with three heated and one cold chains, estimated for 20,000,000 generations, and sampled every 1,000 generations. The first 25% of samples were discarded as burn-in and the post-burn-in samples were summarized into a 50% majority-rule consensus tree. We assessed parameter stationarity by determining that the standard deviation of split frequencies has reached below 0.005 by the end of the run. We also used Tracer v. 1.7.1 (Rambaut et al. 2018) to ensure the effective sample size (ESS) for each parameter was above 200. For the ML tree, we used raxmlGUI v. 2.0 (Edler et al. 2021) and implemented 1,000 rapid bootstrap replicates with parameter and branch length optimizations. The trees output from the BI and ML analyses were visualized in FigTree v. 1.4.4 (http://tree.bio.ed.ac.uk/software/figtree/). We considered posterior probability (PP) ≥ 0.95 for the BI tree and bootstrap support value (BS) ≥ 95% for the ML tree as indicators of strongly supported relationships.

### ﻿Range estimation

We generated ecological niche models (ENMs) to estimate the geographic distributions of the three *Gloydius* species within DPR Korea. As there are no reliable species occurrence points recorded within the nation (i.e., occurrence points with accurate longitude/latitude coordinates), we collected the occurrence points recorded from adjacent nations (R Korea, Russia, and PR China) to generate the models. First, we downloaded the occurrence points of the three target *Gloydius* species from the Global Biodiversity Information Facility (GBIF) using the R package *megaSDM* (Shipley et al. 2022; downloaded on 19 August 2022). Next, we mapped the GBIF occurrence points in ArcGIS Pro v. 2.6.0 (ESRI, Redlands, CA) to verify the positional consistency between the downloaded occurrence points and the known distributions of each species. This process revealed a large number of *G.intermedius* occurrence points from R Korea with dubious spatial accuracy, with many occurrence points falling outside the known distribution and habitat requirements for the species (Jang et al. 2016; Lee et al. 2023). Such inconsistencies in the occurrence data suggested a large number of misidentifications at the species level or erroneous data entries and we removed them from the dataset. Therefore, for *G.intermedius*, we directly downloaded research-grade occurrence data from iNaturalist (www.inaturalist.org; accessed on 27 November 2023). The iNaturalist data enabled us to directly verify the species-level identification using the photographs attached to each observation. In addition, we retrieved additional *Gloydius* occurrence points from R Korea recorded during the 4^th^ National Ecosystem Surveys (www.nie-ecobank.kr/cmmn/Index.do; Kim et al. 2021). Thus, we collected a total of 3551 occurrence points for *G.ussuriensis*, 424 for *G.brevicauda*, and 184 for *G.intermedius*. Next, we spatially rarefied the occurrence points using the R package *humboldt* (Brown and Carnaval 2019). Considering the number and spatial clustering of the occurrence data, we applied a rarefying distance of 20 km to *G.ussuriensis* and a rarefying distance of 1 km for *G.brevicauda* and *G.intermedius*. The final dataset used for modeling consisted of 331 occurrence points for *G.ussuriensis*, 373 for *G.brevicauda*, and 154 for *G.intermedius*.

For the environmental inputs, we considered climatic, topographic, and land cover variables to broadly characterize the habitat conditions of the three *Gloydius* species. We obtained the 19 bioclimatic variables from WorldClim 2.1 (www.worldclim.org; Fick and Hijmans 2017) and a forest cover layer from EarthEnv (www.earthenv.org; Tuanmu and Jetz 2014). For topographic variables, we obtained the elevation raster from WorldClim 2.1 and also derived a slope layer from the elevation layer using ArcGIS. All the environmental layers were in 1 km spatial resolution (= 0.008333 dd) and cropped to the geographic extent covering the occurrence points of all three *Gloydius* species. From the initial set of 22 environmental layers, we conducted a Pearson’s correlation test to remove highly correlated variables (| *r* | > 0.7). Therefore, a total of nine variables were retained for niche modeling: annual mean temperature (Bio 1), mean diurnal range (Bio 2), isothermality (Bio 3), mean temperature of wettest quarter (Bio 8), annual precipitation (Bio 12), precipitation of wettest month (Bio 13), precipitation seasonality (Bio 15), forest cover, and slope. The variable selection step was conducted using the *ntbox* package (Osorio-Olvera et al. 2020).

The abundance of *Gloydius* occurrence points in R Korea and the absence of occurrence points in DPR Korea created a significant spatial sampling bias. Therefore, we used target group background sampling (Kramer-Schadt et al. 2013; Merow et al. 2013) to compensate for this bias. We collected a total of 61,576 occurrence points for 440 reptile species distributed across the full geographic extent of the study area using the R package *megaSDM* (Shipley et al. 2022). We then spatially rarefied this dataset using a 1 km distance parameter and converted it into a kernel density raster using the *MASS* package (Venables and Ripley 2002) and sampled 10,000 background points from this density raster.

We implemented the maximum entropy (MaxEnt; Phillips et al. 2017) algorithm in the *SDMtune* package (Vignali et al. 2020) to generate ENMs for the three *Gloydius* species. For each species, we tested the combinations of six MaxEnt feature classes (L, LQ, H, LQH, LQHP, LQHPT) and regularization values ranging from 0.5 to 5 in increments of 0.5. We evaluated the candidate models based on a random 10-fold cross-validation. From a set of 60 models tested for each species, we calculated the area under the receiver operating characteristic curve (AUC) from the training (AUC_TRAIN_) and testing data (AUC_TEST_). We then selected the optimal hyperparameter combinations based on the highest AUC_TEST_ and minimum AUC_DIFF_ (AUC_TRAIN_ – AUC_TEST_; Warren and Seifert 2011), thereby selecting a model with high predictive ability and a low degree of overfitting. The optimal model for *G.ussuriensis* was made with LQHP features combined with a regularization of 1.5, the optimal model for *G.brevicauda* was made with LQHP features and a regularization of 0.5, and the optimal model for *G.intermedius* was made with LQHPT features and a regularization of 1.0. In addition to AUC_TEST_ and AUC_DIFF_, we calculated the True Skill Statistic (TSS; Allouche et al. 2006) as an additional statistical metric to evaluate the final selected models. We also visually examined the output predictions to ensure the overall consistency between the model predictions and known geographic distributions. We generated response curves of the fitted models and assessed the variable importance by calculating permutation importance.

The model outputs were in complementary log-log transformation (cloglog; Phillips et al. 2017) format and we masked the prediction maps to the boundaries of DPR Korea. We also calculated the maximum training sensitivity plus specificity cloglog threshold (MTSS) to convert the continuous prediction outputs into binary presence/absence maps. These binary maps were also used to calculate the total area of suitable habitats within DPR Korea estimated from the niche models. The ecological niche modeling and associated analyses were conducted in R v. 4.2.2 (R Core Team 2022).

## ﻿Results

### ﻿Morphological identification

We were able to identify the four *Gloydius* specimens from DPR Korea based on a combination of scalation and body pattern characteristics. The voucher 19GNK001 had 21 midbody dorsal scale rows, 46 subcaudal scales, and 147 ventral scales. The body pattern of this specimen consisted of a series of inconspicuous circular spots, many of which were joined in the middle. The voucher 19GNK002 had 22 midbody dorsal scale rows, 43 subcaudal scales, and 152 ventral scales, with body patterns consisting of conspicuous and irregular crossbands. The voucher 19GNK003 had 21 midbody dorsal scale rows, 32 subcaudal scales, and 144 ventral scales, with a conspicuous yellow tail tip, black venter with white markings, and body patterns consisting of dark brown circular spots. The voucher 19GNK004 had 21 midbody dorsal scale rows, 43 subcaudal scales, and 156 ventral scales, with body patterns consisting of inconspicuous circular spots. Based on these characteristics, we identified vouchers 19GNK001 and 19GNK004 as *G.ussuriensis*, voucher 19GNK002 as *G.intermedius*, and voucher 19GNK003 as *G.brevicauda*. See below for a detailed description of each specimen.

**Figure 3.**
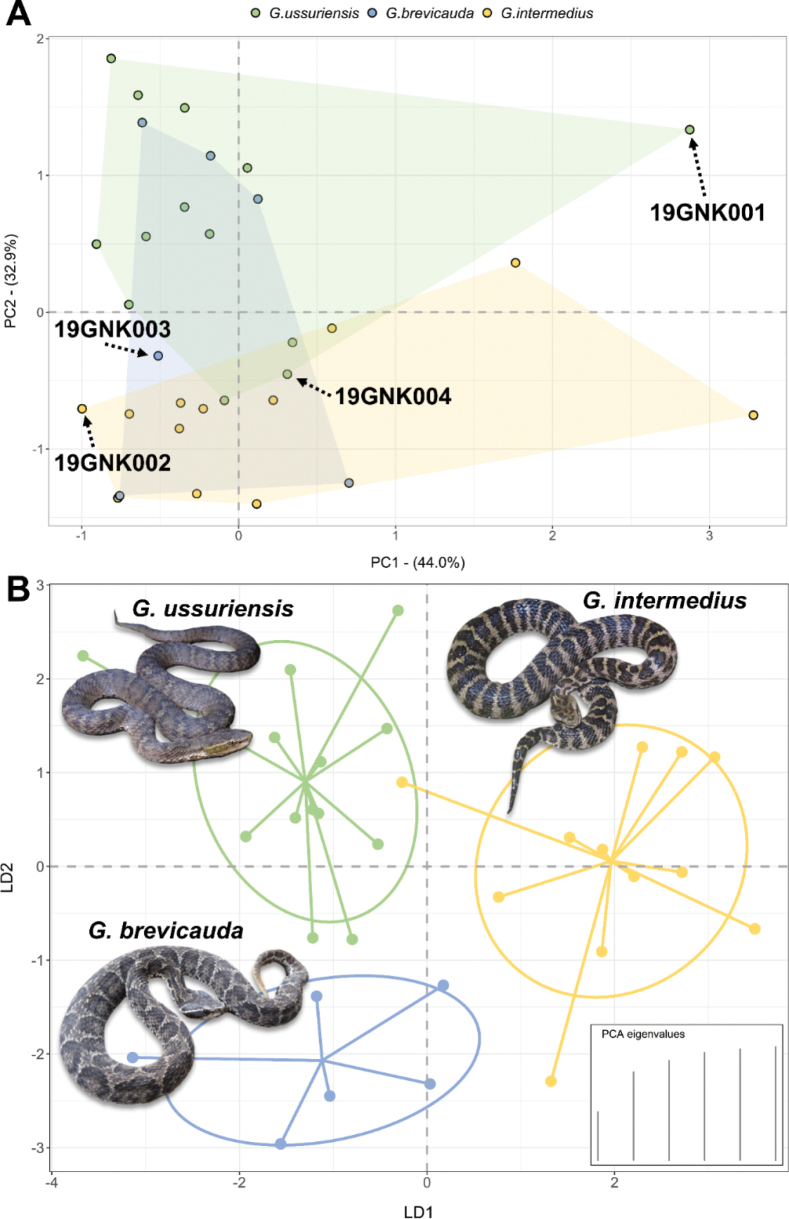
The results of the multivariate morphological analyses to identify *Gloydius* specimens from DPR Korea. A. The result of a principal components analysis (PCA) on 11 morphological characters. Note the broad overlap between the three species within the morphological space defined by the first two principal components. The location of each specimen from DPR Korea in this morphological space is indicated with a black arrow; B. The result of a discriminant analysis of principal components (DAPC). The three clusters correspond to the three species of *Gloydius* examined in this study. One *G.intermedius* sample that is located within the *G.ussuriensis* cluster corresponds to the heavily distorted voucher 19GNK002 from DPR Korea.

The results of the multivariate morphological analyses with added comparative materials also identified three distinct clusters corresponding to the three species (Fig. 3). Based on the results of PCA, the first two PCs explained 76.86% of the variance within our morphological data. The ratio of interorbital distance to total body length and the ratio of head width to total body length had the highest contributions to PC1 and the total body length and head width contributed the most to PC2. The three species showed broad overlaps within the morphological space defined by the first two PCs (Fig. 3A). On the other hand, the results of the DAPC showed three distinct clusters corresponding to three *Gloydius* species (Fig. 3B). The vouchers 19GNK001 and 19GNK004 were identified as *G.ussuriensis*, consistent with the initial morphological identification based on scalation and body patterns. The voucher 19GNK003 was identified as *G.brevicauda*, also consistent with the initial morphological identification. However, the voucher 19GNK002 clustered with *G.ussuriensis*, although the scalation and body patterns provided an unambiguous identification of this specimen as *G.intermedius*.

### ﻿Phylogenetic analyses

**Figure 4.**
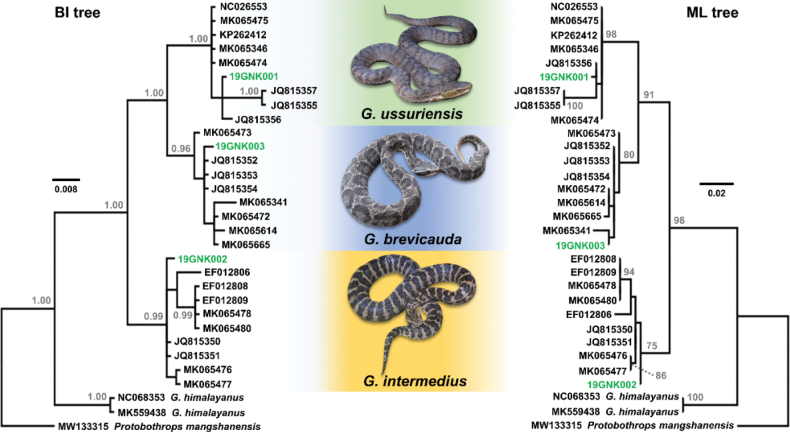
The Bayesian Inference (BI; left panel) and Maximum Likelihood (ML; right panel) phylogenies of the three *Gloydius* species from northeast Asia, based on the 351 bp fragment of mitochondrial 12S ribosomal RNA (12S rRNA) gene. The samples originating from DPR Korea are highlighted with green text. The node values for the BI tree indicate posterior probability (PP) > 0.95, and the node values for the ML tree indicate bootstrap support value (BS) > 70%. The monophyly of each *Gloydius* species is generally well supported in both trees, and each of the three samples from DPR Korea is nested in the clade corresponding to each of the three *Gloydius* species. The trees were visualized in FigTree v. 1.4.4.

The phylogeny inferred from the partial 12S rRNA gene fragment provided sufficient resolution to identify the three specimens from DPR Korea at the species level. Both BI and ML trees recovered monophyletic *Gloydius* and recovered a strongly supported clade composed of *G.ussuriensis* + *G.brevicauda* + *G.intermedius* (PP = 1.00; BS = 98%; Fig. 4). Both trees recovered three well-supported clades each corresponding to *G.ussuriensis* (PP = 1.00; BS = 98%), *G.brevicauda* (PP = 0.96; BS = 80%), and *G.intermedius* (PP = 0.99; BS = 75%). In both trees, the voucher 19GNK001 was clustered within the *G.ussuriensis* clade, 19GNK003 was clustered within the *G.brevicauda* clade, and 19GNK002 was clustered within the *G.intermedius* clade.

As both BI and ML trees produced essentially similar topologies, we present the results primarily based on the BI tree below, while noting any considerable topological disagreements between the ML and BI trees. For the *G.ussuriensis* clade, the five samples from China occupied the basal position in a polytomy with respect to the four samples from the Korean Peninsula. The sample from DPR Korea (19GNK001) was clustered with a sample from Incheon, two samples from Jeju, and a sample from Sacheon, R Korea (Fig. 4). In the *G.brevicauda* clade, a single specimen from Liaoning, China, was at the base of the clade, and the specimen 19GNK003 from DPR Korea was grouped with three samples from R Korea in a polytomy. Additionally, this grouping of the Korean samples was positioned basal to a subclade containing four samples from China. Notably, for the ML tree, the sample from DPR Korea was clustered with a sample from Jiangsu, China, rather than clustering with samples from R Korea (Fig. 4). Regarding the *G.intermedius* clade, the sample from DPR Korea (19GNK002) was in a polytomy at a basal node with two samples from R Korea. This clade also contained two resolved subclades composed of five samples from northeastern China and another grouping composed of two samples from Shaanxi, China. However, in the ML tree, the sample from DPR Korea was basal to all other samples. Although there are polytomies in all the species clades without a high resolution of geographical structuring, the recovered relationships among the species unambiguously supported the initial species identification of our focal specimens based on morphological data.

### ﻿Range estimation

The ENMs for each species generally had adequate predictive performances and a low degree of overfitting. The model evaluation metrics for each species were as follows: *G.ussuriensis* (AUC_TEST_ = 0.692; TSS = 0.340; AUC_DIFF_ = 0.028), *G.brevicauda* (AUC_TEST_ = 0.718; TSS = 0.361; AUC_DIFF_ = 0.033), and *G.intermedius* (AUC_TEST_ = 0.856; TSS = 0.598; AUC_DIFF_ = 0.041). The output prediction maps for the entire study extent are in Suppl. material 2.

For the *G.ussuriensis* model, the most important variable was precipitation seasonality (Bio 15), followed by isothermality (Bio 3), mean temperature of the wettest quarter (Bio 8), slope, annual mean temperature (Bio 1), mean diurnal range (Bio 2), forest cover, annual precipitation (Bio 12), and the precipitation of wettest month (Bio 13). For *G.brevicauda*, the most important variable was precipitation seasonality (Bio 15), followed by annual precipitation (Bio 12), annual mean temperature (Bio 1), mean diurnal range (Bio 2), precipitation of wettest month (Bio 13), forest cover, slope, isothermality (Bio 3), and the mean temperature of the wettest quarter (Bio 8). For *G.intermedius*, the most important variable was precipitation seasonality (Bio 15), followed by isothermality (Bio 3), annual mean temperature (Bio 1), annual precipitation (Bio 12), slope, forest cover, mean diurnal range (Bio 2), mean temperature of the wettest quarter (Bio 8), and the precipitation of wettest month (Bio 13). The permutation importance values for each variable per species are in Suppl. material 1, and the response curves for each variable are in Suppl. material 3.

**Figure 5.**
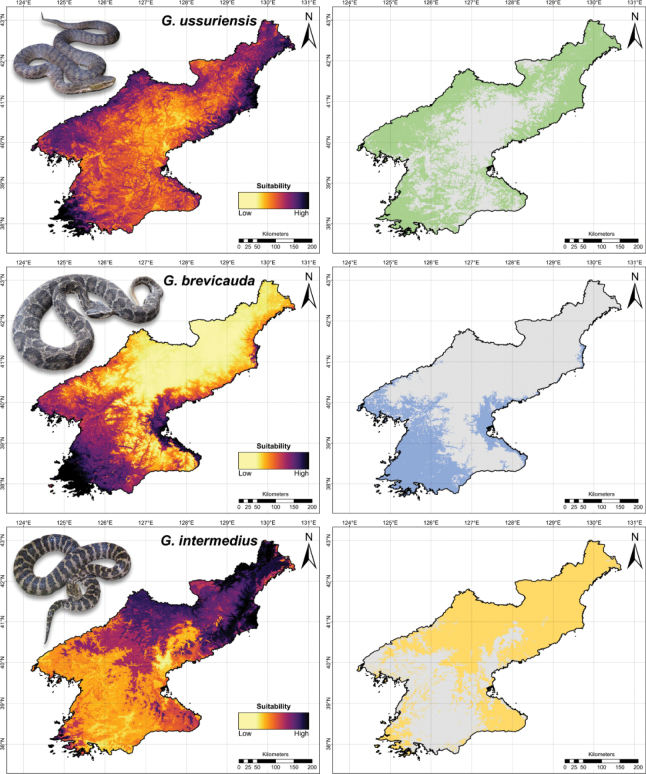
The maximum entropy (MaxEnt) ecological niche model predictions for the three *Gloydius* species found in northeast Asia. The continuous prediction outputs are on the left panel and the binary maps are on the right panel. The maximum training sensitivity plus specificity cloglog threshold (MTSS) was used to convert the continuous outputs into binary maps. The maps were visualized in ArcGIS Pro v. 2.6.0 using the WGS 84 geographic projection.

For *G.ussuriensis*, the niche model predicted intermediate to high habitat suitability across DPR Korea, and the binary map estimated 68,979 km^2^ of suitable habitats across low to high-elevation regions (Fig. 5). On the other hand, the niche model for *G.brevicauda* predicted highly suitable habitats primarily in the western and eastern lowland regions of DPR Korea, whereas the high-elevation regions in northern DPR Korea had low habitat suitability. The binary map predicted 36,511 km^2^ of suitable habitats for *G.brevicauda* within DPR Korea (Fig. 5). For *G.intermedius*, the niche model predicted highly suitable habitats along the eastern and northern mountains of DPR Korea, and the binary map estimated a total of 73,584 km^2^ of suitable habitats (Fig. 5).

### ﻿Description of specimens

#### ﻿ Gloydius ussuriensis

Taxon classificationAnimaliaSquamataViperidae

(Emelianov, 1929)

B24949C6-3666-5803-A516-2EEFA3FCF2A2

Figs 6 , 7

##### Referred specimens.

Vouchers 19GNK001 and 19GNK004, deposited in Nanjing Forestry University, PR China.

##### Identification.

The species-level identification of the specimens is based on diagnostic scale counts, body patterns, multivariate morphological analyses, comparisons to the live individuals of the same species (Fig. 1A, B) and confirmed by molecular analyses.

##### Color in preservation (19GNK001).

The specimen shows considerable discoloration, and the characteristic coloration of *G.ussuriensis* is not well-preserved. The dorsal part of the specimen is greyish-brown in color. A series of inconspicuous circular patterns are present along the length of the body. The patterns are separated at the anterior portion of the body but gradually merge in the posterior half of the body. When viewed from the lateral side, the postocular bands are dark brown in coloration. The supra- and infralabials are immaculate and have light brown/pale orange coloration. The ventrals are light brown/pale orange in coloration and largely immaculate in the anterior half of the body, and small dark brown spots are visible on the posterior half of the body.

**Figure 6.**
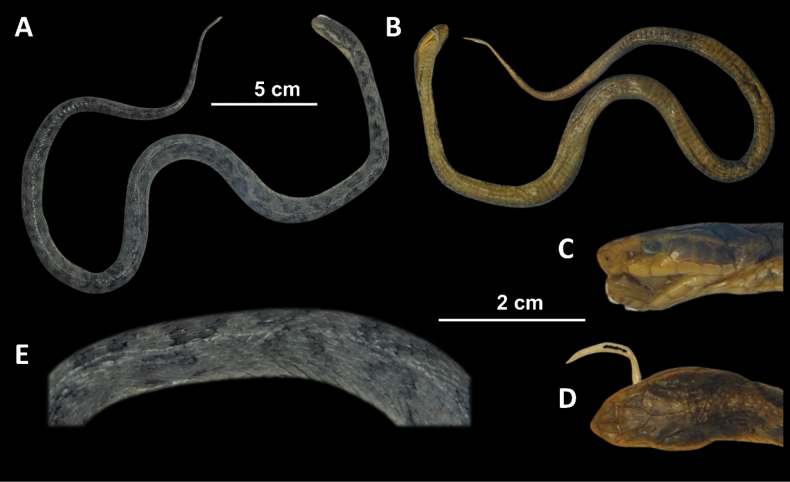
*Gloydiusussuriensis* voucher 19GNK001, collected from Rajin, DPR Korea photographed from A. Dorsal and B. Ventral angles; C. Close-up of the left lateral side of the head; D. Close-up of the dorsal surface of the head; E. Close-up of the dorsal surface at mid-body.

##### Scalation and morphology (19GNK001).

Supralabials 7/7 (left/right). Infralabials 10/10 (left/right). Midbody dorsal scale rows 21. Ventral scales 147. Subcaudal scales 46. The following morphological measurements are given in millimeters (mm). TOL 482 mm. SVL 408 mm. TAL 74 mm. HL 16.9 mm. HW 11.5 mm. HH 9.4 mm. ED 2.5 mm. IOD 7.4 mm.

**Figure 7.**
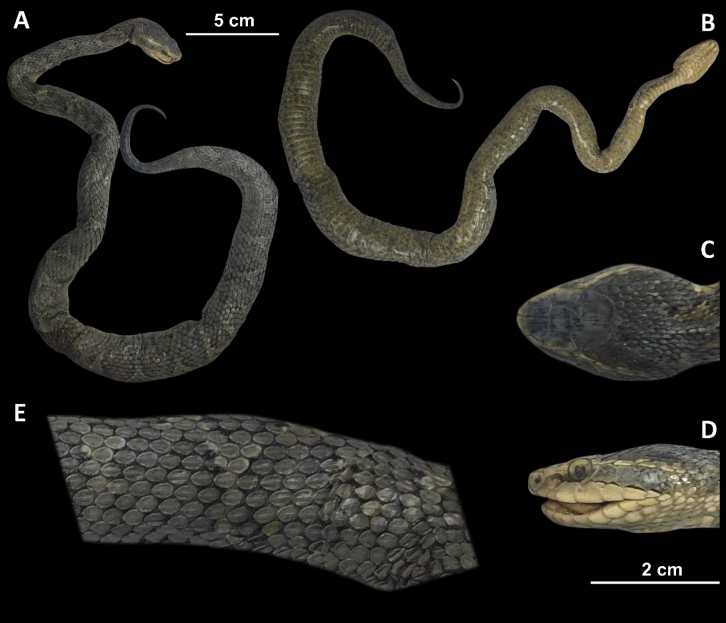
*Gloydiusussuriensis* voucher 19GNK004, collected from Gaesong, DPR Korea photographed from A. Dorsal and B. Ventral angles; C. Close-up of the dorsal surface of the head; D. Close-up of the left lateral side of the head; E. Close-up of the dorsal surface at mid-body.

##### Color in preservation (19GNK004).

The specimen shows considerable discoloration, and the characteristic coloration of *G.ussuriensis* is not well-preserved. The dorsal coloration of the specimen is brownish-grey. A series of inconspicuous circular patterns are present along the length of the body, most of which are joined in the middle to form irregular band-like patterns. When viewed from the lateral side, the postocular bands are pale brownish-grey in color. The supra- and infralabials are immaculate and light beige in color. The ten anteriormost ventrals are immaculate and beige in coloration. However, small black and dark grey markings are visible on the remaining ventrals. The ventral coloration gradually changes from beige to dark brownish-grey with the increase in the number and size of dark-colored markings.

##### Scalation and morphology (19GNK004).

Supralabials 7/7 (left/right). Infra­labials 10/10 (left/right). Midbody dorsal scale rows 21. Ventral scales 156. Subcaudal scales 43. The following morphological measurements are given in millimeters (mm). TOL 574 mm. SVL 499 mm. TAL 75 mm. HL 26.4 mm. HW 16.4 mm. HH 10.3 mm. ED 3.5 mm. IOD 8.7 mm.

#### ﻿ Gloydius brevicauda

Taxon classificationAnimaliaSquamataViperidae

(Stejneger, 1907)

BA230400-F797-5A4E-830A-835782B98CD1

Fig. 8

##### Referred specimen.

Voucher 19GNK003, deposited in Nanjing Forestry University, PR China.

##### Identification.

The species-level identification of the specimen is based on diagnostic scalations, coloration, multivariate morphological analyses, comparisons to the live individuals of the same species (Fig. 1C, D), and corroborated by molecular analyses.

##### Color in preservation.

The original body coloration typical of *G.brevicauda* is largely intact, with some discoloration in the posterior 2/3 of the specimen. The dorsal coloration is greyish-brown, with conspicuous circular patterns present along the length of the body. When viewed from the lateral side, conspicuous black postocular bands are present. The supra- and infralabials are cream-colored, with the first four infralabials and the last three supralabials on each side of the head having black markings. The ventral side of the head is mottled with black markings. The anterior ventrals are mostly cream-colored with black mottled patterns. The black coloration on the ventrals expands gradually toward the posterior half of the body, where the ventral scales are almost entirely black with some white mottling.

**Figure 8.**
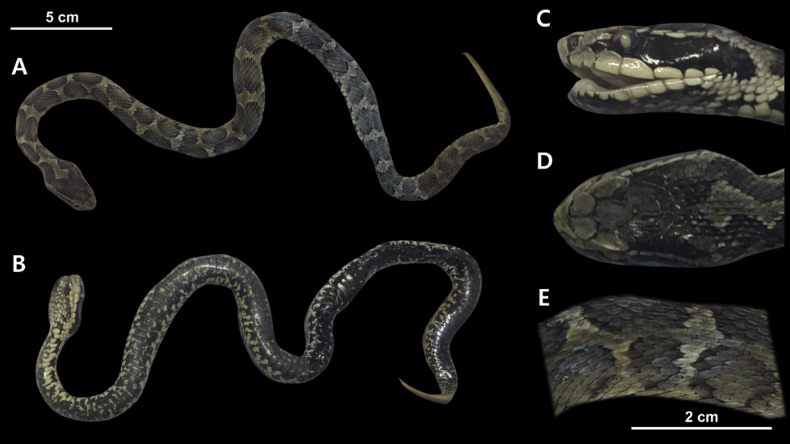
*Gloydiusbrevicauda* voucher 19GNK003, collected from Gaesong, DPR Korea photographed from A. Dorsal and B. Ventral angles; C. Close-up of the left lateral side of the head; D. Close-up of the dorsal surface of the head; E. Close-up of the dorsal surface at mid-body.

##### Scalation and morphology.

Supralabials 7/7 (left/right). Infralabials 10/10 (left/right). Midbody dorsal scale rows 21. Ventral scales 144. Subcaudal scales 32. The following morphological measurements are given in millimeters (mm). TOL 474 mm. SVL 426 mm. TAL 48 mm. HL 28.2 mm. HW 15.4 mm. HH 9.2 mm. ED 3.5 mm. IOD 10.2 mm.

#### ﻿ Gloydius intermedius

Taxon classificationAnimaliaSquamataViperidae

(Strauch, 1868)

8A9FEFF4-5711-553B-B4B1-9D5D12A2C75A

Fig. 9

##### Referred specimen.

19GNK002, deposited in Nanjing Forestry University, PR China.

##### Identification.

The species-level identification of the specimen is based on diagnostic scalation, coloration, multivariate morphological analyses, comparisons to the live individuals of the same species (Fig. 1E, F), and corroborated by molecular analyses.

##### Color in preservation.

The specimen is significantly discolored, and the characteristic coloration of *G.intermedius* is not well-preserved. The head shape is also considerably distorted from preservation. The dorsal coloration of the specimen is pale bluish-grey. At least 21 irregular crossbands are clearly visible. However, it is difficult to count the total number of crossbands due to discoloration in the posterior half of the specimen. Unlike vouchers 19GNK001, 19GNK003, and 19GNK004, distinct postocular bands are not present. Overall, the supra- and infralabials have beige coloration with small dark brown spots. Except for the anteriormost portion, the ventral surface of the specimen is largely greenish-grey in color. There are black markings running down the center of the ventral scales. These markings expand in the posterior 2/3 of the body, where the ventral surface appears almost black in color.

**Figure 9.**
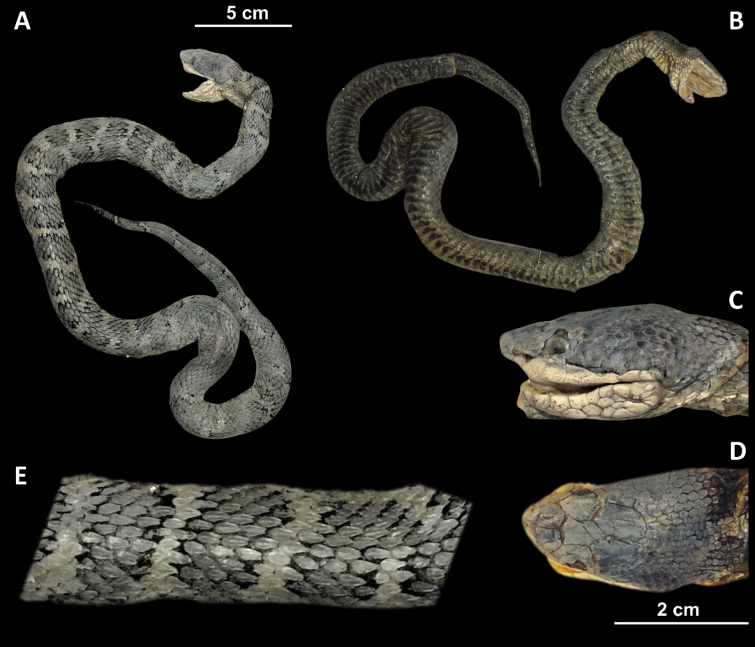
*Gloydiusintermedius* voucher 19GNK002, collected from an unspecified location in DPR Korea photographed from A. Dorsal and B. Ventral angles; C. Close-up of the left lateral side of the head; D. Close-up of the dorsal surface of the head; E. Close-up of the dorsal surface at mid-body.

##### Scalation and morphology.

Supralabials 8/7 (left/right). Infralabials 10/10 (left/right). Midbody dorsal scale rows 21. Ventral scales 156. Subcaudal scales 43. The following morphological measurements are given in millimeters (mm). TOL 605 mm. SVL 518 mm. TAL 87 mm. HL 29.7 mm. HW 19.1 mm. HH 14.4 mm. ED 3.5 mm. IOD 9.9 mm.

## ﻿Discussion

Our study represents a major contribution to the knowledge of *Gloydius* found in DPR Korea, a nation that has been largely inaccessible for field research. Through visual examination of specimens, scale counts, comparisons with live specimens, and multivariate analyses, we identified three different species of *Gloydius* from the specimens retrieved from “snake liquors.” We corroborated this initial identification through phylogenetic analyses and unambiguously identified the presence of at least three *Gloydius* species within DPR Korea. In addition, we generated predictive distribution maps of the three *Gloydius* species using ENMs, greatly augmenting the existing literature-based information on their geographic distributions in DPR Korea. The morphological, genetic, and distribution data for *Gloydius* generated from this study have not been available previously and will contribute to our broader understanding of the herpetofauna in DPR Korea specifically and of northeast Asia generally.

In his book on the herpetofauna of DPR Korea, Won (1971) cited various subspecies and species designations provided by Stejneger (1907) and Pope (1935). However, he considered the evidence for these designations insufficient and subsequently treated all pitvipers found in the nation as a single species under the common name “viper” (살모사; *Ancistrodonhalys*). This treatment as a single species does not allow species-level identification using measurement data and scalation characteristics provided therein. However, other morphological differences between specimens collected in different habitat types are mentioned. For example, Won (1971) described the “lowland form” as possessing broad black postocular bands with thin and distinct yellowish-white stripes above the postocular bands. The “highland form,” on the other hand, is described as possessing either indistinct postocular bands or postocular bands with variable shapes. Considering the ecology of *Gloydius* species in the adjacent R Korea (Do and Nam 2021; Lee et al. 2023) and comparing the descriptions of Won (1971) to the specimens examined in this study, the “lowland form” is most likely *G.brevicauda* while the “highland form” likely encompasses both *G.intermedius* and *G.ussuriensis*.

In a more recent description of pitvipers from DPR Korea, Kim and Han (2009) divided the “viper” (*Ancistrodonhalys*) of Won (1971) into three distinct species: “viper” (살모사; *Agkistrodonhalys* (Pallas, 1776)), “black viper” (검은살모사; *Agkistrodonblomhoffii* (Stejneger, 1907)), and “rock viper” (돌살모사; *Agkistrodonsaxatilis* (Emelianov, 1937)). This split is based on scale counts, body coloration and patterns, and “molecular differences based on gel electrophoresis” (details on molecular differences were not provided). Kim and Han (2009) also provided hand-drawn black-and-white illustrations for each species. While the illustrations are not detailed, it is possible to make species-level identification based on ecological characteristics and scalation provided with each species account.

For example, the “rock viper” is described as having a dark or yellowish brown body coloration and having indistinct postocular bands. This species is also noted for having a considerably larger body size than the other two species, a habitat preference for higher-elevation regions (500 ~ 2300 m asl), and a dietary preference for rodents. Based on these characteristics and the scientific name used (*A.saxatilis*), this species is most likely *G.intermedius* (Orlov and Barabanov 1999). The counts of midbody dorsal scale rows (21–23), ventral scales (145–160), and subcaudal scales (37–45), as well as comparisons with the specimen 19GNK002 and photographs of live individuals, are consistent with this species being *G.intermedius* (Orlov and Barabanov 1999; Koo et al. 2017; Lee et al. 2023; Uetz et al. 2024). Our ENM prediction also suggests that the suitable habitats of *G.intermedius* in DPR Korea are mostly located along high-elevation mountains.

On the other hand, the “black viper” is described as having dark brown or yellowish-brown dorsal body coloration and black or yellowish-brown ventral coloration with black or dark brown patterns. In addition, the presence of black postocular bands and thin yellowish-white stripes above them is noted. The species is described as having a dietary preference for anurans and habitat preferences for agricultural landscapes, grasslands, and streams in lowland areas below 300 m asl. The species is also described as being generally larger than the “viper” *Agkistrodonhalys*. It is not possible to make a species-level identification based on the name *Agkistrodonblomhoffii* and synonyms used (*Ancistrodonb.ussuriensis* and *Agkistrodonb.brevicaudus*). However, based on the combination of morphological and ecological characteristics described in the text, this species is most likely *G.brevicauda*. The counts of midbody scale rows (20–21), ventral scales (136–142), subcaudal scales (30–42), and comparisons with the specimen 19GNK002 as well as with the photographs of live individuals are consistent with identification as *G.brevicauda* (Koo et al. 2017; Lee et al. 2023; Uetz et al. 2024). Our ENM for *G.brevicauda* also predicted suitable habitats within the low-elevation regions of DPR Korea.

The treatment of “viper” (*Agkistrodonhalys*) in Kim and Han (2009) differs somewhat from that of Won (1971). In Kim and Han (2009), the “viper” is described as having variable body coloration ranging from dark to reddish- or yellowish-brown. The body patterns are also described as being highly variable, ranging from “wave-like” irregular crossbands to indistinct ring-like patterns. In contrast to the “black viper,” this species is described as having a relatively small head and slender body. This species is described as having dietary preferences for fish, anurans, small rodents, and birds and inhabiting mountains, abandoned agricultural fields, and rock piles adjacent to them. The reported elevational distribution of this species is 100–1500 m asl. It is not possible to make a species-level identification based on the name (*Agkistrodonhalys*) or synonyms used (*Agkistrodonblomhoffiibrevicaudus* (Stejneger, 1907), *Agkistrodonhalysbrevicaudus* (Nikolsky, 1916), and *Trigonocephalusblomhoffii* (Boie, 1826)). Based on the provided morphological and ecological descriptions, however, this particular species is most likely to be *Gloydiusussuriensis*. The counts of midbody dorsal scale rows (19–21), ventral scales (140–155), and subcaudal scales (30–50) are consistent with the identification as *G.ussuriensis* (Koo et al. 2017; Lee et al. 2023; Uetz et al. 2024). Our ENM for *G.ussuriensis* also predicted a broad area of suitable habitats across DPR Korea, consistent with the reported elevational range.

Our study significantly builds on the species accounts of *Gloydius* reported by Won (1971) and Kim and Han (2009) by providing morphological and molecular data. Even with considerable discoloration observed in most of the specimens examined here, a combination of body patterns and scalation was sufficient to initially identify these specimens to the species level. The result of the DAPC also supported initial morphological identification based on body patterns and scalation. While the *G.intermedius* voucher 19GNK002 was misclassified as *G.ussuriensis* by the DAPC, this is most likely due to the distortion in the head of this specimen. The molecular data corroborated our initial identifications based on external morphology. Mitochondrial 12S rRNA sequences extracted from the “snake liquor” specimens were short fragments (351 bp), likely due to preservation in low-concentration (~ 16–25%) ethanol and subsequent degradation of DNA. While the size of our genetic dataset is limited by the availability of complementary sequences and did not resolve finer scale relationships within clades, phylogenetic analyses were nevertheless sufficient to identify these specimens to the species level. While we were only able to sequence a single mitochondrial gene fragment due to degradation, next-generation sequencing approaches may yield additional loci from these degraded specimens (Ruane and Austin 2017; Bernstein and Ruane 2022) for phylogenomic analyses.

Predictive distribution maps generated from ENMs also provide useful insights into the geographic distributions of these species in DPR Korea. The distributions reported by Won (1971) and Kim and Han (2009) were at coarse geographic scales (county- or province-level) and did not permit their application to spatial analyses. The ENMs and range maps generated in this study can be utilized to assess the conservation status of these species and predict the influence of future environmental changes on their distributions (Ahmadi et al. 2019; Shin et al. 2021b; Do et al. 2022). As all three species are commonly found for sale as snake liquor, none of them is likely to be critically endangered in DPR Korea, a point to contrast with *Viperaberus* (Linnaeus, 1758), which we have never seen used for snake liquor. Furthermore, the range maps can be overlaid with the human population data to assess the potential risk of snakebite envenomings in DPR Korea. Similar approaches have been applied to various nations to identify the hotspots of snakebite envenomings (Yañez-Arenas et al. 2016; Zacarias and Loyola 2019; Yousefi et al. 2020). Assessment of snakebite risk in this way can provide critical data for information “grey-zones” like DPR Korea to better estimate the global snakebite burden.

## Supplementary Material

XML Treatment for Gloydius ussuriensis

XML Treatment for Gloydius brevicauda

XML Treatment for Gloydius intermedius

## ﻿Additional information

### Author ORCIDs

Yucheol Shin https://orcid.org/0000-0002-4606-2907

Siti N. Othman https://orcid.org/0000-0002-3894-7553

Dallin B. Kohler https://orcid.org/0000-0003-2951-3371

Irina Maslova https://orcid.org/0000-0002-6240-3812

Kevin R. Messenger https://orcid.org/0000-0002-5363-7393

Amaël Borzée https://orcid.org/0000-0003-1093-677X

### Data availability

All of the data that support the findings of this study are available in the main text or Supplementary Information.

## Supplementary materials

Supplementary material 1 The importance of environmental variables used for MaxEnt modeling according to permutation importance Data type pdf Explanation note The permutation importance value for the most important variable for each species is highlighted in bold. https://binary.pensoft.net/file/1397598This dataset is made available under the Open Database License (http://opendatacommons.org/licenses/odbl/1.0/). The Open Database License (ODbL) is a license agreement intended to allow users to freely share, modify, and use this Dataset while maintaining this same freedom for others, provided that the original source and author(s) are credited.Yucheol Shin, Siti N. Othman, Dallin Kohler, Irina Maslova, Kevin R. Messenger, Amaël Borzée

Supplementary material 2 The continuous MaxEnt model predictions for the three Gloydius species across the entire model calibration extent Data type png Explanation note The maps were visualized in ArcGIS Pro v. 2.6.0 using the WGS 84 geographic projections. https://binary.pensoft.net/file/1397599This dataset is made available under the Open Database License (http://opendatacommons.org/licenses/odbl/1.0/). The Open Database License (ODbL) is a license agreement intended to allow users to freely share, modify, and use this Dataset while maintaining this same freedom for others, provided that the original source and author(s) are credited.Yucheol Shin, Siti N. Othman, Dallin Kohler, Irina Maslova, Kevin R. Messenger, Amaël Borzée

Supplementary material 3 MaxEnt response curves for *G.ussuriensis*, *G.brevicauda*, and *G.intermedius* Data type png Explanation note MaxEnt response curves for *G.ussuriensis* (occurrence *n* = 331), *G.brevicauda* (occurrences *n* = 373), and *G.intermedius* (occurrences *n* = 154). The full variable names for the bioclimatic variables are as follows: annual mean temperature (Bio 1), mean diurnal range (Bio 2), isothermality (Bio 3), mean temperature of wettest quarter (Bio 8), annual precipitation (Bio 12), precipitation of wettest month (Bio 13), precipitation seasonality (Bio 15). https://binary.pensoft.net/file/1397600This dataset is made available under the Open Database License (http://opendatacommons.org/licenses/odbl/1.0/). The Open Database License (ODbL) is a license agreement intended to allow users to freely share, modify, and use this Dataset while maintaining this same freedom for others, provided that the original source and author(s) are credited.Yucheol Shin, Siti N. Othman, Dallin Kohler, Irina Maslova, Kevin R. Messenger, Amaël Borzée
