# Supplementary material for: ﻿Contributions to the knowledge of pitvipers (Viperidae, Gloydius) in the Democratic People’s Republic of Korea: identification, description of specimens, and geographical distribution
Source: Zookeys. 2025 Aug 19;1249:193–221. doi: 10.3897/zookeys.1249.142916 (PMC12381584; doi:10.3897/zookeys.1249.142916)
Supplement: Supplementary material 1 — The importance of environmental variables used for MaxEnt modeling according to permutation importance [file zookeys-1249-193_article-142916__-s001.pdf]

Table S1. The importance of environmental variables used for MaxEnt modeling according to permutation importance. The permutation importance value for the most important variable for each species is highlighted in bold.

**Variables**	**Species**		
	G. ussuriensis	*G. brevicauda*	*G. intermedius*
Annual mean temperature (Bio 1)	9.71	16.05	12.53
Mean diurnal range (Bio 2)	7.52	13.20	2.34
Isothermality (Bio 3)	17.39	2.09	30.27
Mean temperature of wettest quarter (Bio 8)	13.32	1.97	1.92
Annual precipitation (Bio 12)	3.84	18.45	10.51
Precipitation of wettest month (Bio 13)	0.10	12.07	1.21
Precipitation seasonality (Bio 15)	**29.66**	**24.66**	**30.89**
Forest cover	6.71	7.10	4.32
Slope	11.73	4.40	6.00
