# Supplementary figures and images for: ﻿Contributions to the knowledge of pitvipers (Viperidae, Gloydius) in the Democratic People’s Republic of Korea: identification, description of specimens, and geographical distribution
Source: Zookeys. 2025 Aug 19;1249:193–221. doi: 10.3897/zookeys.1249.142916 (PMC12381584; doi:10.3897/zookeys.1249.142916)

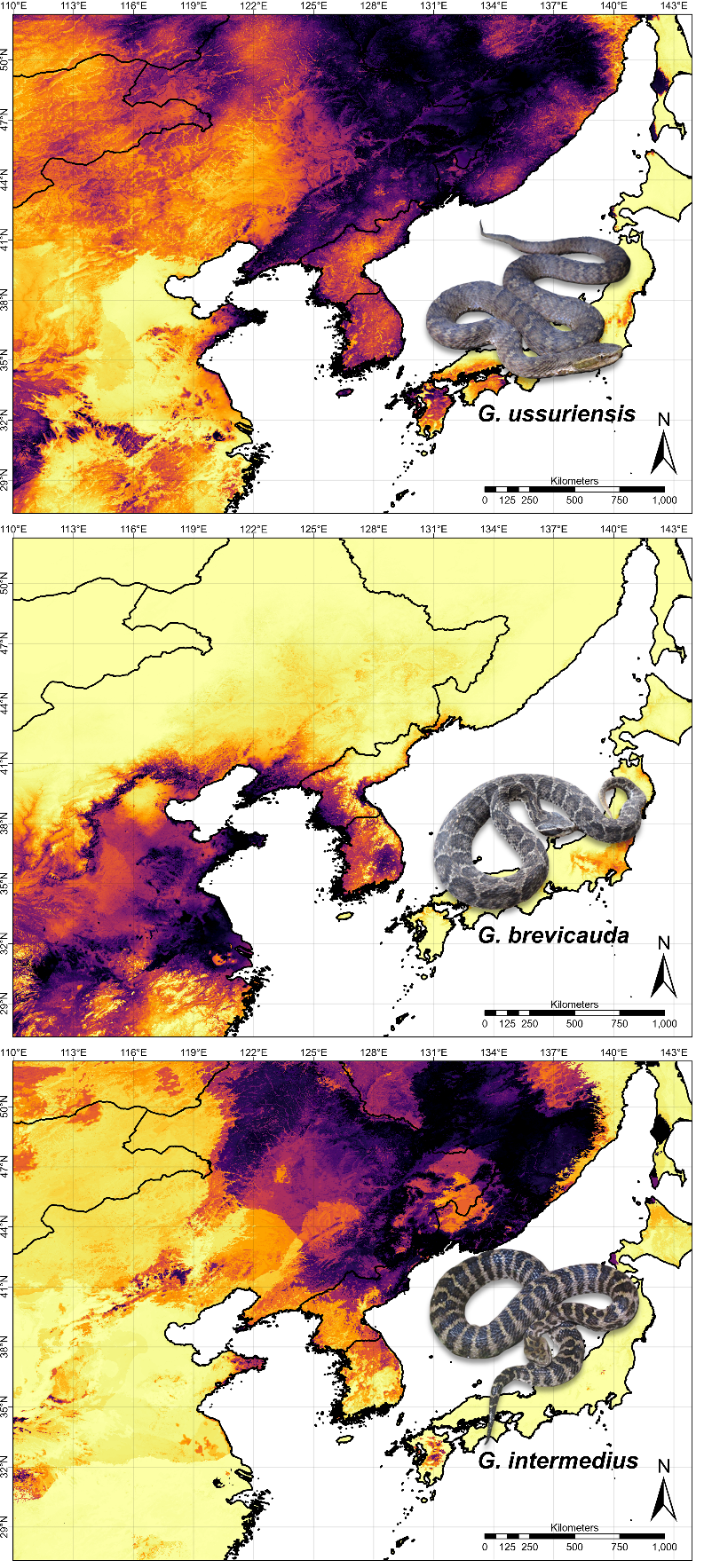

Supplement: Supplementary material 2 — The continuous MaxEnt model predictions for the three Gloydius species across the entire model calibration extent [file zookeys-1249-193_article-142916__-s002.png]

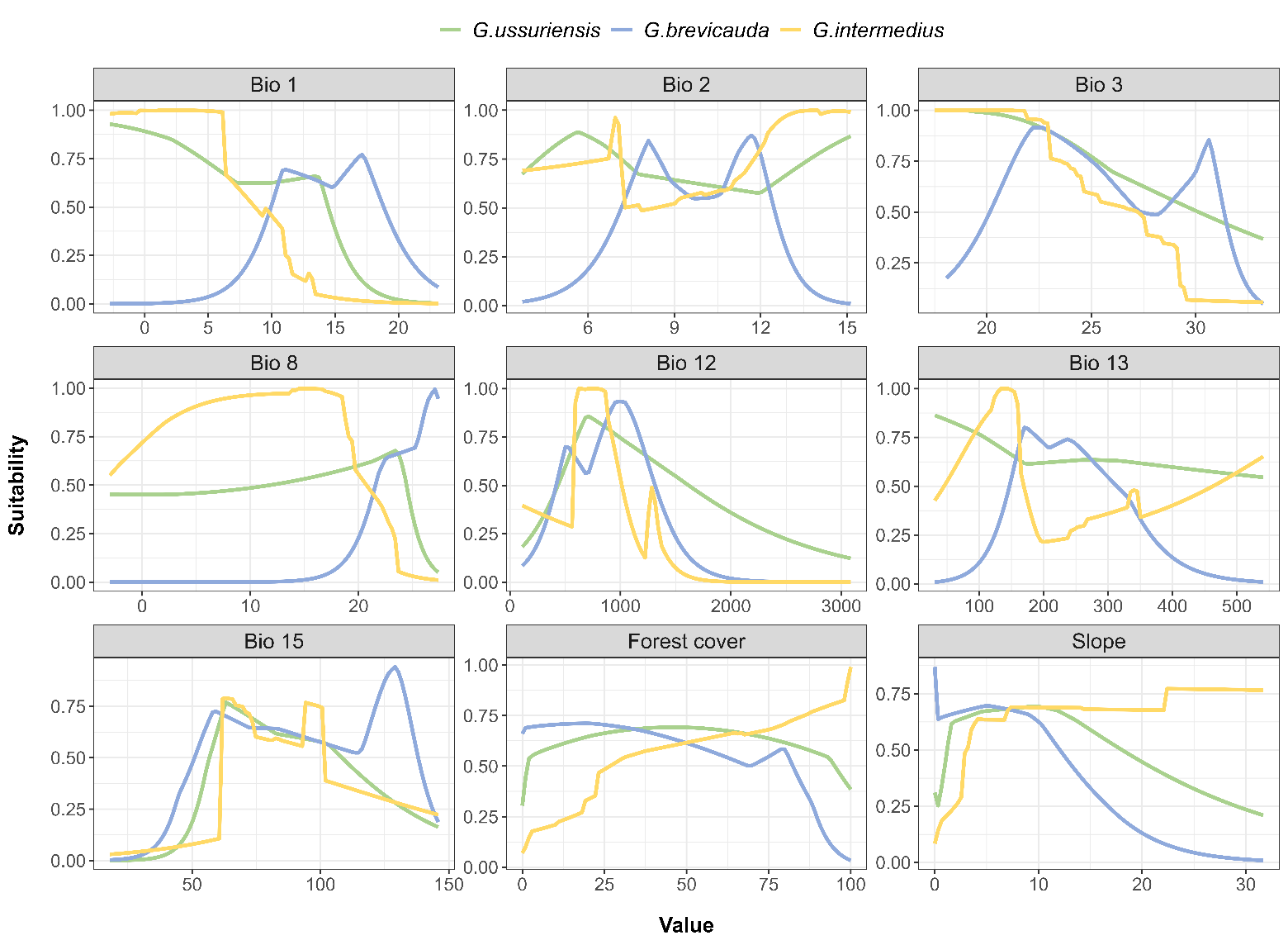

Supplement: Supplementary material 3 — MaxEnt response curves for G.ussuriensis, G.brevicauda, and G.intermedius [file zookeys-1249-193_article-142916__-s003.png]
